# Omega-3 fatty acid supplement use and oxidative stress levels in pregnancy

**DOI:** 10.1371/journal.pone.0240244

**Published:** 2020-10-23

**Authors:** Erin G. Sley, Emma M. Rosen, Thomas J. van ‘t Erve, Sheela Sathyanarayana, Emily S. Barrett, Ruby H. N. Nguyen, Nicole R. Bush, Ginger L. Milne, Shanna H. Swan, Kelly K. Ferguson

**Affiliations:** 1 Epidemiology Branch, National Institute of Environmental Health Sciences, Research Triangle Park, North Carolina, United States of America; 2 Department of Maternal and Child Health, University of North Carolina, Chapel Hill, North Carolina, United States of America; 3 Department of Pediatrics, Seattle Children’s Research Institute, University of Washington, Seattle, Washington, United States of America; 4 Department of Environmental and Occupational Health Sciences, University of Washington, Seattle, Washington, United States of America; 5 Department of Epidemiology, Rutgers School of Public Health, Piscataway, New Jersey, United States of America; 6 Department of Epidemiology & Community Health, University of Minnesota, Minneapolis, Minnesota, United States of America; 7 Departments of Psychiatry and Pediatrics, University of California at San Francisco, San Francisco, California, United States of America; 8 Division of Clinical Pharmacology, Vanderbilt University School of Medicine, Nashville, Tennessee, United States of America; 9 Department of Preventive Medicine, Icahn School of Medicine at Mount Sinai, New York, New York, United States of America; University of Mississippi Medical Center, UNITED STATES

## Abstract

Oxidative stress is a biological imbalance in reactive oxygen species and antioxidants. Increased oxidative stress during pregnancy has been associated with adverse birth outcomes. Omega-3 fatty acid (n-3 FA) supplementation may decrease oxidative stress; however, this relationship is seldom examined during pregnancy. This study assessed the association between n-3 FA supplement use during pregnancy and urinary oxidative stress biomarker concentrations. Data came from The Infant Development and the Environment Study (TIDES), a prospective cohort study that recruited pregnant women in 4 US cities between 2010–2012. Third trimester n-3 FA intake was self-reported. Third trimester urinary 8-iso-prostaglandin F_2α_ (8-iso-PGF_2α_) was measured as an oxidative stress biomarker. Additionally, we measured the major metabolite of 8-iso-PGF_2α_ and Prostaglandin F_2α_ (PGF_2α_) and utilized the 8-iso-PGF_2α_ to PGF_2α_ ratio to calculate the change in 8-iso-PGF_2α_ reflecting oxidative stress versus inflammation. Adjusted linear models were used to determine associations with control for confounding. Of 725 women, 165 reported n-3 FA supplement use in the third trimester. In adjusted linear models, n-3 FA use was associated with 10.2% lower levels of 8-iso-PGF_2α_ (95% Confidence Interval [CI]: -19.6, 0.25) and 10.3% lower levels of the metabolite (95% CI: -17.1, -2.91). No associations were observed with PGF_2α_. The lower levels of 8-iso-PGF_2α_ appeared to reflect a decrease in oxidative stress (percent change with supplement use: -18.7, 95% CI: -30.1, -5.32) rather than inflammation. Overall, third trimester n-3 FA intake was associated with lower concentrations of 8-iso-PGF_2α_ and its metabolite, suggesting a decrease in maternal oxidative stress during pregnancy.

## Introduction

Oxidative stress, a biological imbalance in reactive oxygen species and antioxidant levels, results in an excess of free chemical radicals that can cause lipid peroxidation and other outcomes leading to cell damage [1]. In pregnancy, elevated oxidative stress levels have been associated with prevalent adverse birth outcomes in the United States, including placental aging [2], intrauterine growth restriction [3, 4], preterm labor [5–8], and preeclampsia [9, 10]. These outcomes have been associated with increased risks of morbidity and mortality for mothers and infants [11]. An accessible method to lower oxidative stress levels during pregnancy could help prevent these adverse birth outcomes.

Omega-3 fatty acid (n-3 FA) supplementation has the potential to reduce damage caused by oxidative stress. One possible biological mechanism by which this may occur is through the actual replacement of arachidonic acid with n-3 FA in cell membranes. Then, when an excess of reactive oxygen species causes oxidation of lipids in those membranes, the cleaved products of n-3 FA are hypothesized to be less damaging to other cells than those of arachidonic acid [12, 13].

A number of studies have examined the relationship between n-3 FA supplementation and oxidative stress in humans [14–16]. However, only two of these studies have evaluated associations with maternal oxidative stress levels during pregnancy [17, 18]. In one randomized trial of pregnant women with gestational diabetes, women who were given n-3 FA supplements in pregnancy had decreased concentrations of plasma malondialdehyde (MDA), a biomarker of oxidative stress, compared to those who did not [17]. Another trial evaluated this association in healthy pregnancies by measuring maternal plasma thiobarbituric acid-reactive substance (TBARS) levels as an indicator of oxidative stress, and observed a surprising positive association [18]. Of note, these trials were restricted by smaller samples sizes (N = 54–270) and limitations in oxidative stress biomarker measurement [19–23]. Specifically, MDA has been considered unreliable [20] while TBARS assays are often non-specific and results may represent other processes aside from lipid peroxidation [24]. Both biomarkers are known to be susceptible to cross-reactions with other existing biochemicals [20].

We sought to examine the association between prenatal n-3 FA supplementation and maternal oxidative stress using data from The Infant Development and Environment Study (TIDES), a multi-center US pregnancy cohort. We examined the relationship between maternal self-reported n-3 FA supplement use and urinary concentrations of 8-iso-PGF_2α_, its major metabolite (metabolite 2,3-dinor-5,6-dihydro-15-F_2_T-isoprostane), and Prostaglandin F_2α_ (PGF_2α_). These well-established biomarkers of oxidative stress are direct products of lipid peroxidation that represent lipid damage through specific pathways, are stable during pregnancy, and easily detectible in urine [6, 8, 25, 26]. Furthermore, we used the novel 8-iso-PGF_2α_/ PGF_2α_ ratio to investigate whether changes in 8-iso-PGF_2α_ were attributable to either upregulation of inflammatory pathways or to true oxidative stress [27]. Thus, utilizing these biomarkers represents a significant improvement on previous research on this relationship in pregnant women.

## Materials and methods

### Study population

TIDES is a multi-center prospective cohort of women enrolled during pregnancy [28, 29]. Women were recruited between August 2010 and August 2012 at clinics in the University of California, San Francisco, CA; University of Rochester Medical Center, NY; University of Minnesota, MN; and University of Washington-Seattle Children’s Hospital, WA. Cohort eligibility included a minimum age of 18 years, the ability to read and write in English, and a maximum gestational age of 13 weeks at the time of recruitment, to reflect first trimester exposures in relation to neonatal outcomes. At three prenatal appointments, targeted at one per trimester, participants completed questionnaires and provided urine samples (protocols described in detail elsewhere) [29]. TIDES was approved by the institutional review board at each study site and each participant provided signed informed consent prior to data collection.

All questionnaire data was collected at each of the four study sites and is held centrally at the Icahn School of Medicine at Mount Sinai. Use of this data was deemed exempt by the NIEHS IRB, and was transferred to NIEHS under a data use agreement between the two institutions. The oxidative stress biomarker data was generated at Vanderbilt University Medical Center and transferred directly to NIEHS.

The present analysis was a cross-sectional assessment of the associations between n-3 FA supplementation, as reported by questionnaire, and urinary oxidative stress biomarker concentrations. From the overall study population we included women who provided a urine sample and responded to the n-3 FA survey question at the third study visit. From the overall study population (N = 971), these restrictions excluded 210 women who were missing a urine sample at the third visit and 36 women who did not respond to the n-3 FA survey question, for a final sample size of N = 725.

### n-3 FA supplement use

Participants completed self-administered questionnaires at each visit to provide information on lifestyle, demographic characteristics, and health. Supplement use was assessed with a list; participants were asked to check a box next to any supplement that they had consumed daily for at least one consecutive week in their current trimester. The list included n-3 FA supplements, listed as “fish oil supplements”, prenatal vitamins, multivitamins, and other individual supplements.

### Biomarkers of oxidative stress

Biomarkers of oxidative stress were measured in third visit urine samples at the Vanderbilt Eicosanoid Core Laboratory. Samples were collected at a mean of 32.6 weeks gestation (range = 25.7–41.1 weeks gestation). Samples were analyzed via gas chromatography-negative ion chemical ionization mass spectrometry for several compounds derived from arachidonic acid: 8-iso-PGF_2α_; its primary metabolite 2,3-dinor-5,6-dihydro-15-F_2_T-isoprostane; and PGF_2α_. The metabolite of 8-iso-PGF_2α_ may be more sensitive than the parent compound; it is generated in the lungs rather than the kidneys and thus may be a better indicator of oxidative stress occurring throughout the entire body [30]. PGF_2α_ is a reliable marker of inflammation [31].

8-iso-PGF_2α_ can be generated through upregulation of inflammatory pathways or through chemical oxidative stress [27]. To distinguish the source in our study, we used the novel 8-iso-PGF_2α_/ PGF_2α_ ratio which evaluates the proportion of 8-iso-PGF_2α_ produced from enzymatic synthesis by prostaglandin-endoperoxide synthases (PGHS) (i.e., inflammation) as compared to chemical lipid peroxidation (i.e., oxidative stress). Higher levels of the ratio indicate a greater contribution to 8-iso-PGF_2α_ from chemical lipid peroxidation, i.e., oxidative stress, as compared to enzymatic synthesis, i.e., inflammation, and it can be used to calculate the proportion of 8-iso-PGF_2α_ originating from each source [27]. This ratio has been examined and validated in other studies as well [32–34]. Thus, we examined a total of five markers: three that were measured (8-iso-PGF_2α_, 8-iso-PGF_2α_ metabolite, PGF_2α_) and two that were derived (8-iso-PGF_2α_ chemical and 8-iso-PGF_2α_ enzymatic).

For examining distributions of oxidative stress biomarkers, we corrected each concentration for urinary specific gravity because this correction has been found suitable in accounting for the hydration status of pregnant women when analyzing urinary biomarkers. Compared to creatine, specific gravity correction is more reproducible within-person with less systematic variance [35]. We used the following formula for correction: O_c_ = O[(1.014–1]/Sg– 1]] where O_c_ represents the specific gravity-corrected oxidative stress biomarker concentration, O represents the measured biomarker concentration, 1.014 is the median specific gravity of all TIDES samples, and Sg is the specific gravity level measured in that sample. For statistical models, raw concentrations were modeled and specific gravity was included as a covariate.

### Statistical analysis

All statistical analyses were performed using SAS 9.4 (Cary, NC]. First, we examined demographic characteristics associated with n-3 FA use using chi-squared tests. We then assessed associations between n-3 FA consumption and urinary oxidative stress biomarker concentrations. Finally, we created crude and adjusted linear models to evaluate the associations between n-3 FA intake and each oxidative stress marker. For all statistical models, urinary oxidative stress biomarker concentrations were natural log-transformed. Crude models included gestational age at urine sample collection and specific gravity as covariates. For adjusted models, confounders were identified using a Directed Acyclic Graph developed following a literature review. Potential confounders were then empirically evaluated within our dataset for their impact on effect estimates. Final models included gestational age at sample collection (weeks, continuous], maternal age (years, continuous), specific gravity (continuous), race (White/Black/other), education (college degree vs. none), and study center. Additional variables such as income, smoking status, prenatal vitamin use, pre-pregnancy BMI and fish intake were considered but not included due to low sample size or little influence on effect estimates (less than 10% change). All effect estimates and 95% confidence intervals (CIs) were scaled to present the percent change in oxidative stress biomarker in association with n-3 FA supplementation in pregnancy, for interpretability.

### Sensitivity analyses

We ran several additional analyses to test the robustness of our findings. Because the decision to take n-3 FA is strongly associated with socioeconomic status, we examined models stratified by education level [36]. Second, because individuals in our sample who supplement with n-3 FA were more likely to take prenatal vitamins which may also influence oxidative stress levels as well, we restricted our analytic population to women who reported prenatal vitamin use in the third trimester. Use of other vitamin supplementation was uncommon in our population. Of the 725 women in our study, the most common supplements, aside from n-3 FA, were vitamin D (n = 148), iron (n = 141), and calcium (n = 96). Other individual supplements, including amino acids, herbal supplements, and vitamins A, C, E or K were used much less frequently (n = 1–6). These compounds were not associated with oxidative stress biomarkers in preliminary analyses and thus were not examined in subsequent models.

## Results

Our study included 725 women with urinary oxidative stress biomarker measurements and n-3 FA supplement questionnaire responses at the third study visit. Participants were primarily White, married or living with their partner, and did not smoke or drink alcohol during pregnancy (Table 1). On average, the women were 31.7 years old and had a pre-pregnancy BMI of 25.6 kg/m^2^. In our sample, 165 (23%) women reported taking an n-3 FA supplement for at least a week in the third trimester. These women were more likely to be White, non-smokers, and married or living with their partner, compared to those who did not take n-3 FA supplements. Women who took n-3 FA supplements were also older and had higher incomes and education levels then women who did not take n-3 FA supplements. Specific gravity was higher in women who did not use N-3 supplements, reflecting more concentrated urine. The median (IQR) in users was 1.011 (1.007, 1.016) and 1.015 (1.009, 1.021) in non-users.

**Table 1:** Demographic characteristics of The Infant Development and Environment Study (TIDES) cohort stratified by n-3 FA supplement use in the third trimester (n = 725).

Characteristic	n-3 FA Supplementation (n = 165) n (%)	No n-3 FA Supplementation (n = 560) n (%)
Age (years)		
<25	1 (0.6)	93 (17.4)
25–29	31 (19.4)	120 (22.5)
30–34	58 (36.3)	187 (35.0)
≥35	70 (43.8)	134 (25.1)
Pre-pregnancy BMI (kg/m^2^)		
<18.5	3 (1.8)	12 (2.2)
18.5–24.99	110 (66.7)	301 (54.3)
25–29.99	32 (19.4)	121 (21.8)
≥30	20 (12.1)	120 (21.7)
Race		
White	136 (82.4)	360 (64.4)
Black/African American	4 (2.4)	91 (16.3)
Other	25 (15.2)	108 (19.3)
Smoking		
None	158 (100)	509 (93.6)
Any	0 (0)	35 (6.4)
Alcohol		
None	140 (88.6)	503 (92.3)
Any	18 (11.4)	42 (7.7)
Marital status		
Living together/married	159 (96.4)	443 (79.4)
Single	6 (3.6)	115 (20.6)
Education		
No college degree	8 (4.9)	180 (32.4)
College degree	157 (95.2)	376 (67.6)
Income		
<25k	10 (6.2)	162 (29.8)
45k-65k	35 (21.7)	107 (19.7)
>65k	116 (72.1)	274 (50.5)
Previous Pregnancies		
0	69 (42.3)	205 (37.0)
1–3	87 (53.4)	283 (51.1)
4–6	7 (4.3)	66 (11.9)
Infant Sex		
Male	91 (56.5)	253 (45.6)
Female	70 (43.5)	302 (54.4)
Study Center		
UCSF	62 (37.6)	128 (22.9)
UMN	65 (39.4)	137 (24.5)
URMC	8 (4.9)	201 (35.9)
UW	30 (18.2)	94 (16.8)
Prenatal Vitamin in 3^rd^ Trimester		
No	1 (0.6)	72 (12.9)
Yes	164 (99.4)	488 (87.1)

BMI, Body Mass Index; UCSF, University of California, San Francisco, CA; UMN, University of Minnesota, MN; URMC, University of Rochester Medical Center, NY; UW, University of Washington-Seattle Children’s Hospital, WA.

Concentrations of both 8-iso-PGF_2α_ and the 8-iso-PGF_2α_ metabolite were lower among women who took n-3 FA in the third trimester compared to those who did not (Table 2). In adjusted models, n-3 FA consumption was associated with 10.2% lower levels of 8-iso-PGF_2α_ (95% confidence interval [CI]: -19.6, 0.25) and 10.3% lower levels of the 8-iso-PGF_2α_ metabolite (95% CI: -17.1, -2.91) (Table 3). We did not observe an association between n-3 FA use and PGF_2α_ levels. When we examined associations with the chemical versus the enzymatic fractions of 8-iso-PGF_2α_, n-3 FA use was associated with 18.7% lower levels in the chemical fraction of 8-iso-PGF_2α_ (95% CI: -30.1, -5.32), reflecting oxidative stress, but was not associated with the enzymatic fraction of 8-iso-PGF_2α,_ reflecting inflammation. Results were greater in magnitude in crude models (Table 3); all covariates included in the model meaningfully changed effect estimates compared to the crude model.

**Table 2:** Median (25^th^, 75^th^ percentile) specific gravity-corrected urinary oxidative stress biomarker concentrations (ng/mL) by omega-3 fatty acid (n-3 FA) supplement use in the third trimester.

	n-3 FA supplement use (n = 165)	No n-3 FA supplement use (n = 560)
Measured		
8-iso-prostaglandin F_2α_	0.83 (0.60, 1.09)	1.00 (0.68, 1.47)
8-iso-prostaglandin F_2α_ metabolite	0.55 (0.44, 0.73)	0.64 (0.49, 0.89)
Prostaglandin F_2α_	2.03 (1.17, 3.54)	2.03 (1.33, 3.21)
Derived^a^		
8-iso-prostaglandin F_2α_, enzymatic	0.31 (0.15. 0.50)	0.28 (0.12, 0.49)
8-iso-prostaglandin F_2α_, chemical	0.45 (0.27, 0.67)	0.64 (0.39, 1.01)

^a^. The enzymatic and chemical fractions of 8-iso-prostaglandin F_2α_ were derived from the 8-iso-PGF_2α_ to PGF_2α_ ratio.

**Table 3:** Adjusted percent change (95% confidence intervals) in urinary oxidative stress levels in association with omega-3 fatty acid supplement use in the 3^rd^ trimester of pregnancy (n = 693).

	Crude Percent Change^a^ (95% CI)	Adjusted Percent Change^b^ (95% CI)
Measured		
8-iso-prostaglandin F_2α_	-20.8 (-29.1, -11.6)	-10.2 (-19.6, 0.25)
8-iso-prostaglandin F_2α_ metabolite	-18.3 (-24.5, -11.6)	-10.3 (-17.1, -2.91)
Prostaglandin F_2α_	-1.75 (-13.9, 12.1)	1.91 (-11.2, 17.0)
Derived		
8-iso-prostaglandin F_2α_, enzymatic	34.8 (-3.20, 87.7)	16.7 (-17.4, 64.8)
8-iso-prostaglandin F_2α_, chemical	-32.0 (-41.6, -20.8)	-18.7 (-30.1, -5.32)

^a^. Model includes gestational age at sample collection and specific gravity.

^b^. Model includes gestational age at sample collection, specific gravity, maternal age (continuous), race (white, black, other), education (college degree vs. none), and study center.

Overall, our sensitivity analyses produced results fairly similar to our primary results. First, in a stratified analysis, there was no evidence of statistical interaction by education level (S1 Table). However, associations were greater in magnitude among women with no college degree. Second, we restricted our sample to women who reported third trimester prenatal vitamin use (n = 622). Effect estimates were similar to those observed in the original analytic sample (S2 Table).

## Discussion

In a healthy cohort of US pregnant women, we found that n-3 FA supplementation was associated with lower levels of 8-iso-PGF_2α_ and its metabolite after controlling for relevant confounders, indicating lower levels of oxidative stress. Our examination of the fractions of 8-iso-PGF_2α_ originating from chemical oxidative stress as compared to inflammation suggested that changes observed were attributable to lower chemical oxidative stress levels. Although these estimates are adjusted for relevant confounders based on substantive and statistical evidence, these results should be interpreted with caution as additional, unmeasured health behaviors may influence this association.

Overall, randomized trials primarily suggest that n-3 FAs are effective in reducing oxidative stress in both animals and humans [14–16, 37–40]. Although, one study has suggested that prolonged use in animals may actually increase oxidative stress levels [41]. Previous animal and human studies that have explored the association between prenatal n-3 FA supplementation and maternal oxidative stress in pregnancy have been inconclusive. Most, but not all, findings from animal studies align with our results that n-3 FA supplementation during gestation is associated with lower maternal oxidative stress biomarker concentrations [42–44]. The two previous human studies that have assessed this relationship in pregnant women have produced mixed results [17, 18]. Our results align with those from Jamilian et al. who found that n-3 FA supplementation was associated with decreased oxidative stress levels in women with gestational diabetes [17]. Finally, although not directly related, this evidence is also consistent with studies that have examined associations between prenatal n-3 FA supplementation and cord blood or neonatal oxidative stress levels [45–48]. Animal studies have also observed that maternal n-3 FA supplementation is associated with lower levels of oxidative stress biomarkers in placenta and offspring [42–44, 49–54].

Previous studies of n-3 FA supplementation in pregnancy were limited by the sample sizes and characteristics of their study population as well as the biomarkers used assess oxidative stress, which could explain some of the differences observed. The only other study to evaluate the impact of n-3 FA supplementation on oxidative stress during healthy pregnancy observed an elevation in oxidative stress, as indicated by TBARS levels, in association with fish oil supplementation [18]. However, TBARS has a number of limitations as a biomarker of oxidative stress. TBARS assays can be unreliable in providing direct measures of lipid peroxidation. These assays are sensitive to cross-reactions with other compounds, which can lead to inaccurate results [20, 24]. Another study which assessed associations between n-3 FA supplementation and oxidative stress in pregnant women, but which used seafood portions instead of pill supplements, also had differing findings from ours in that they observed no differences in plasma or urinary measurements of 8-iso-PGF_2α_ levels in the treatment and control groups [55]. However, the differences in results between that study and ours could be attributed to the fact that use of oral supplements likely delivers higher n-3 FA amounts than the seafood portions that were administered. Our findings are more consistent with the aforementioned study of prenatal supplementation and oxidative stress in pregnancy by Jamilian et al., which observed decreased levels in association with supplement use, despite the fact that they also used a less reliable oxidative stress biomarker, malondialdehyde [20, 24].

Our findings additionally add to this body of literature by improving the ability to distinguish the pathway by which n-3 FA supplementation decreases urinary 8-iso-PGF_2α_ concentrations. Previous studies have noted that supplementation is also associated with lower levels of inflammation biomarkers, such as C-reactive protein [16, 17]. Because inflammation can lead to a generation of oxidative stress biomarkers, it can be difficult to distinguish which mechanism is impacted by supplementation in these studies. We did not observe associations with PGF_2α_ in our study population. This compound is produced through enzymatic peroxidation of arachidonic acid by the cyclooxygenase enzymes which are induced under an inflammatory state [31]. Because we observed associations with 8-iso-PGF2α, which is largely produced through chemical lipid peroxidation, but not with PGF_2α_, we interpret our findings to indicate that the associations observed between n-3 FA supplementation and 8-iso-PGF2α are attributable to a decrease in oxidative stress rather than inflammation. Furthermore, we examined associations between n-3 FA supplementation and 8-iso-PGF_2α_ generated from each source, by utilizing a novel ratio of 8-iso-PGF_2α_ and PGF_2α_. These results also showed that n-3 FA supplementation was associated with a change in the chemical fraction of 8-iso-PGF_2α_, which indicates a specific association with oxidative stress.

Elevated levels of oxidative stress biomarkers in pregnancy, including 8-iso-PGF_2α_, have been associated with adverse birth outcomes such as preeclampsia and preterm labor [4, 6]. Thus, theoretically, increased n-3 FA through supplementation could have the potential to mitigate common adverse birth outcomes by reducing oxidative stress levels. However, randomized control trials evaluating the impact of n-3 FA supplements or dietary interventions to increase n-3 FA intake on these outcomes generally report a null effect [56–63]. Examining the association between n-3 FA supplementation and oxidative stress markers may be a more direct way of capturing the biological impact on the body during pregnancy, and could have implications for future studies examining the impact of supplementation on adverse birth outcomes. For example, focusing on this biological mechanism could improve the understanding which of subpopulations (e.g., demographic groups or individuals with co-morbidities] are more or less responsive to the effects of supplementation. It could also provide insight on co-exposures or supplements that either exacerbate or detract from the physiologic effects of supplementation.

Our study used a cross-sectional design to assess associations between n-3 FA supplementation and oxidative stress biomarker levels. However, this approach may be ideal for this research question. n-3 FA levels rise quickly within the body upon consumption of n-3 FA supplements [64] and 8-iso-PGF2α is a direct reflection of lipid peroxidation at the time it is measured [25, 26]. Therefore, it was important that both exposure and outcome measurements were captured at the same timepoint. It should be carefully noted, however, that associations observed in the third trimester may not be generalizable to the rest of pregnancy. Maternal plasma n-3 FA levels generally decrease throughout gestation as a result of increased maternal transfer of n-3 FAs to the fetus [65]. Therefore, the impact of supplementation on maternal oxidative stress levels may be lesser in this time period than in non-pregnant women or in other trimesters.

Our study had several limitations related to assessing n-3 FA. First, we did not have biomarkers for FA status. Thus, we assessed n-3 FA supplementation by questionnaire, and self-report could have led to recall bias and consequently exposure misclassification. However, we would such bias to be non-differential with regard to outcome. Recall bias would most likely be nondifferential because questionnaires were administered in close proximity to time of supplementation intake, and participants did not know their oxidative stress levels [66]. Second, we only had information on whether the participant had taken a n-3 FA supplement for one week during the third trimester and not information on which week; supplementation within the last 1–2 weeks would have been the most relevant [67]. Third, we were unable to assess supplement dosage, which could have led to a lack of precision in our effect estimates and limits our ability to comment on whether or not intake was consistent with current n-3 FA dietary recommendations for pregnant women [68]. Fourth, we were unable to assess dietary patterns or consumption of specific foods, such as fish intake, which are also important sources of n-3 FA. While dietary sources of n-3 FA and other micronutrients could influence oxidative stress levels, we would not expect this to confound our results since it is not apparent that n-3 FA supplementation is associated with this type of dietary intake [69]. Lastly, n-3 supplementation is strongly influenced by demographic factors and health behaviors, some of which were unmeasured in TIDES. Thus, residual confounding is possible and our results should be interpreted with caution.

Our study also had many strengths. We utilized 8-iso-PGF_2α_ and its major metabolite, which are well-established biomarkers of oxidative stress that are direct products of lipid peroxidation and are stable during pregnancy [6, 25]. These markers are also easily detectible in urine, minimally influenced by fasting or diurnal fluctuations and stable over pregnancy [6, 25]. Additionally, we were better able to assess the mechanism underpinning the association between n-3 FA supplementation and 8-iso-PGF_2α_ levels by application of the 8-iso-PGF_2α_/ PGF_2α_ ratio. Finally, our sizable study population, larger than most other studies assessing this association, drew from 4 diverse city study sites across the US allows for considerable generalizability of our findings.

## Conclusions

We observed lower levels of 8-iso-PGF_2α_ and its primary metabolite in association with n-3 FA intake in pregnancy, which were attributable to decreases in chemical oxidative stress. n-3 FA supplementation may be an easily implemented strategy to decrease maternal oxidative stress during pregnancy; however, our results need to be interpreted with caution as residual confounding is possible. Additional research is warranted as these maternal oxidative stress biomarkers have been linked to adverse birth outcomes and n-3 FA supplements are often affordable and easy to obtain. However, the appropriate dose, clinically relevant benefit, and any adverse effects of supplementation need to be more carefully.

## Supporting information

S1 Table Adjusted percent change (95% confidence intervals) in urinary oxidative stress levels in association with omega-3 fatty acid supplement use in the 3^rd^ trimester of pregnancy stratified by education level. a. Model includes gestational age at sample collection, specific gravity, maternal age, race, education, and study center.
(DOCX)

S2 Table Adjusted^a^ percent change (95% confidence intervals) in urinary oxidative stress levels in association with omega-3 fatty acid supplement use in the 3^rd^ trimester, restricted to women who used prenatal vitamins in 3^rd^ trimester. a. Model includes gestational age at sample collection, specific gravity, maternal age, race, education, and study center.
(DOCX)

## References

[1] Neier K, Marchlewicz EH, Dolinoy DC, Padmanabhan V. Assessing human health risk to endocrine disrupting chemicals: a focus on prenatal exposures and oxidative stress. Endocrine Disruptors. 2015;3(1):e1069916-1–e-7. 27231701 10.1080/23273747.2015.1069916 PMC4876868

[2] Sultana Z, Maiti K, Aitken J, Morris J, Dedman L, Smith R. Oxidative stress, placental ageing-related pathologies and adverse pregnancy outcomes. American Journal of Reproductive Immunology. 2017;77(5):1–10. 10.1111/aji.12653 28240397

[3] Biri A, Bozkurt N, Turp A, Kavutcu M, Himmetoglu O, Durak I. Role of oxidative stress in intrauterine growth restriction. Gynecologic and obstetric investigation. 2007;64(4):187–92. 10.1159/000106488 17664879

[4] Mert I, Oruc AS, Yuksel S, Cakar ES, Buyukkagnici U, Karaer A, et al Role of oxidative stress in preeclampsia and intrauterine growth restriction. The journal of obstetrics and gynaecology research. 2012;38(4):658–64. 10.1111/j.1447-0756.2011.01771.x 22380678

[5] Allen KG, Harris MA. The role of n-3 fatty acids in gestation and parturition. Experimental biology and medicine (Maywood, NJ). 2001;226(6):498–506. 10.1177/153537020122600602 11395920

[6] Ferguson KK, Chen YH, VanderWeele TJ, McElrath TF, Meeker JD, Mukherjee B. Mediation of the Relationship between Maternal Phthalate Exposure and Preterm Birth by Oxidative Stress with Repeated Measurements across Pregnancy. Environmental health perspectives. 2017;125(3):488–94. 10.1289/EHP282 27352406 PMC5332184

[7] Menon R. Oxidative stress damage as a detrimental factor in preterm birth pathology. Frontiers in immunology. 2014;5:567–81. 10.3389/fimmu.2014.00567 25429290 PMC4228920

[8] Ferguson KK, McElrath TF, Chen YH, Loch-Caruso R, Mukherjee B, Meeker JD. Repeated measures of urinary oxidative stress biomarkers during pregnancy and preterm birth. Am J Obstet Gynecol. 2015;212(2):208.e1–8. 10.1016/j.ajog.2014.08.007 25111586 PMC4312513

[9] Bilodeau JF, Qin Wei S, Larose J, Greffard K, Moisan V, Audibert F, et al Plasma F2-isoprostane class VI isomers at 12–18 weeks of pregnancy are associated with later occurrence of preeclampsia. Free radical biology & medicine. 2015;85:282–7. 25998422 10.1016/j.freeradbiomed.2015.05.012 PMC4856520

[10] Mehendale S, Kilari A, Dangat K, Taralekar V, Mahadik S, Joshi S. Fatty acids, antioxidants, and oxidative stress in pre-eclampsia. International journal of gynaecology and obstetrics: the official organ of the International Federation of Gynaecology and Obstetrics. 2008;100(3):234–8. 10.1016/j.ijgo.2007.08.011 17977540

[11] Centers for Disease Control. Picture of America Report: Reproductive Outcomes. 2017.

[12] Calder PC. Omega-3 fatty acids and inflammatory processes. Nutrients. 2010;2(3):355–74. 10.3390/nu2030355 22254027 PMC3257651

[13] Kobayashi N, Barnard RJ, Henning SM, Elashoff D, Reddy ST, Cohen P, et al Effect of altering dietary omega-6/omega-3 fatty acid ratios on prostate cancer membrane composition, cyclooxygenase-2, and prostaglandin E2. Clin Cancer Res. 2006;12(15):4662–70. 10.1158/1078-0432.CCR-06-0459 16899616 PMC3410648

[14] Heshmati J, Morvaridzadeh M, Maroufizadeh S, Akbari A, Yavari M, Amirinejad A, et al Omega-3 fatty acids supplementation and oxidative stress parameters: A systematic review and meta-analysis of clinical trials. Pharmacol Res. 2019;149:104462 10.1016/j.phrs.2019.104462 31563611

[15] Kiecolt-Glaser JK, Epel ES, Belury MA, Andridge R, Lin J, Glaser R, et al Omega-3 fatty acids, oxidative stress, and leukocyte telomere length: A randomized controlled trial. Brain, behavior, and immunity. 2013;28:16–24. 10.1016/j.bbi.2012.09.004 23010452 PMC3545053

[16] Hariri M, Djazayery A, Djalali M, Saedisomeolia A, Rahimi A, Abdolahian E. Effect of n-3 supplementation on hyperactivity, oxidative stress and inflammatory mediators in children with attention-deficit-hyperactivity disorder. Malays J Nutr. 2012;18(3):329–35. 24568073

[17] Jamilian M, Samimi M, Kolahdooz F, Khalaji F, Razavi M, Asemi Z. Omega-3 fatty acid supplementation affects pregnancy outcomes in gestational diabetes: a randomized, double-blind, placebo-controlled trial. J Matern Fetal Neonatal Med. 2016;29(4):669–75. 10.3109/14767058.2015.1015980 25747955

[18] Franke C, Demmelmair H, Decsi T, Campoy C, Cruz M, Molina-Font JA, et al Influence of fish oil or folate supplementation on the time course of plasma redox markers during pregnancy. Br J Nutr. 2010;103(11):1648–56. 10.1017/S0007114509993746 20211038

[19] Nielsen F, Mikkelsen B, Nielsen J, Andersen H, Grandjean P. Plasma malondialdehyde as biomarker for oxidative stress: reference interval and effects of life-style factors. Clinical Chemistry. 1997;7(43):1209–14. 9216458

[20] Khoubnasabjafari M, Ansarin K, Jouyban A. Reliability of malondialdehyde as a biomarker of oxidative stress in psychological disorders. Bioimpacts. 2015;5(3):123–7. 10.15171/bi.2015.20 26457249 PMC4597159

[21] Moore K, Roberts K. Measurement of lipid peroxidation. Free Radical Research. 1998;28(6):659–71. 10.3109/10715769809065821 9736317

[22] Marrocco I, Altieri F, Peluso I. Measurement and Clinical Significance of Biomarkers of Oxidative Stress in Humans. Oxidative Medicine and Cellular Longevity. 2017;2017:6501046 10.1155/2017/6501046 28698768 PMC5494111

[23] Hackett C, Linley-Adams M, Lloyd B, Walker V. Plasma malondialdehyde: a poor measure of in vivo lipid peroxidation. Clin Chem. 1988;34(1):208. 3338169

[24] Ho E, Karimi Galougahi K, Liu CC, Bhindi R, Figtree GA. Biological markers of oxidative stress: Applications to cardiovascular research and practice. Redox Biol. 2013;1:483–91. 10.1016/j.redox.2013.07.006 24251116 PMC3830063

[25] Roberts LJ, Morrow JD. Measurement of F(2)-isoprostanes as an index of oxidative stress in vivo. Free radical biology & medicine. 2000;28(4):505–13. 10719231 10.1016/s0891-5849(99)00264-6

[26] Kadiiska MB, Gladen BC, Baird DD, Germolec D, Graham LB, Parker CE, et al Biomarkers of oxidative stress study II: are oxidation products of lipids, proteins, and DNA markers of CCl4 poisoning? Free Radical Biology & Medicine. 2005;38(6):698–710. 15721980 10.1016/j.freeradbiomed.2004.09.017

[27] Van’t Erve TJ, Lih FB, Jelsema C, Deterding LJ, Eling TE, Mason RP, et al Reinterpreting the best biomarker of oxidative stress: The 8-iso-prostaglandin F2alpha/prostaglandin F2alpha ratio shows complex origins of lipid peroxidation biomarkers in animal models. Free radical biology & medicine. 2016;95:65–73. 26964509 10.1016/j.freeradbiomed.2016.03.001 PMC6626672

[28] Barrett ES, Sathyanarayana S, Janssen S, Redmon JB, Nguyen RH, Kobrosly R, et al Environmental health attitudes and behaviors: findings from a large pregnancy cohort study. Eur J Obstet Gynecol Reprod Biol. 2014;176:119–25. 10.1016/j.ejogrb.2014.02.029 24647207 PMC4001243

[29] Swan SH, Sathyanarayana S, Barrett ES, Janssen S, Liu F, Nguyen RH, et al First trimester phthalate exposure and anogenital distance in newborns. Hum Reprod. 2015;30(4):963–72. 10.1093/humrep/deu363 25697839 PMC4359397

[30] Dorjgochoo T, Gao YT, Chow WH, Shu XO, Yang G, Cai Q, et al Major metabolite of F2-isoprostane in urine may be a more sensitive biomarker of oxidative stress than isoprostane itself. The American journal of clinical nutrition. 2012;96(2):405–14. 10.3945/ajcn.112.034918 22760572 PMC3396448

[31] Ricciotti E, FitzGerald GA. Prostaglandins and inflammation. Arteriosclerosis, thrombosis, and vascular biology. 2011;31(5):986–1000. 10.1161/ATVBAHA.110.207449 21508345 PMC3081099

[32] Rund KM, Heylmann D, Seiwert N, Wecklein S, Oger C, Galano JM, et al Formation of trans-epoxy fatty acids correlates with formation of isoprostanes and could serve as biomarker of oxidative stress. Prostaglandins Other Lipid Mediat. 2019;144:106334 10.1016/j.prostaglandins.2019.04.004 31009766

[33] Van’t Erve TJ, Rosen EM, Barrett ES, Nguyen RHN, Sathyanarayana S, Milne GL, et al Phthalates and Phthalate Alternatives Have Diverse Associations with Oxidative Stress and Inflammation in Pregnant Women. Environ Sci Technol. 2019;53(6):3258–67. 10.1021/acs.est.8b05729 30793895 PMC6487641

[34] Rosen EM, van ’t Erve TJ, Boss J, Sathyanarayana S, Barrett ES, Nguyen RHN, et al Urinary oxidative stress biomarkers and accelerated time to spontaneous delivery. Free Radic Biol Med. 2019;130:419–25. 10.1016/j.freeradbiomed.2018.11.011 30445128 PMC6331226

[35] MacPherson S, Arbuckle TE, Fisher M. Adjusting urinary chemical biomarkers for hydration status during pregnancy. J Expo Sci Environ Epidemiol. 2018;28(5):481–93. 10.1038/s41370-018-0043-z 29880833 PMC8075920

[36] Cowan AE, Jun S, Gahche JJ, Tooze JA, Dwyer JT, Eicher-Miller HA, et al Dietary Supplement Use Differs by Socioeconomic and Health-Related Characteristics among U.S. Adults, NHANES 2011(-)2014. Nutrients. 2018;10(8). 10.3390/nu10081114 PMC6116059 30126136

[37] Iraz M, Erdogan H, Ozyurt B, Ozugurlu F, Ozgocmen S, Fadillioglu E. Brief communication: omega-3 essential fatty acid supplementation and erythrocyte oxidant/antioxidant status in rats. Ann Clin Lab Sci. 2005;35(2):169–73. 15943181

[38] Wiest EF, Walsh-Wilcox MT, Walker MK. Omega-3 Polyunsaturated Fatty Acids Protect Against Cigarette Smoke-Induced Oxidative Stress and Vascular Dysfunction. Toxicol Sci. 2017;156(1):300–10. 10.1093/toxsci/kfw255 28115642 PMC6075584

[39] Firat O, Makay O, Yeniay L, Gokce G, Yenisey C, Coker A. Omega-3 fatty acids inhibit oxidative stress in a rat model of liver regeneration. Ann Surg Treat Res. 2017;93(1):1–10. 10.4174/astr.2017.93.1.1 28706885 PMC5507785

[40] Nyby MD, Matsumoto K, Yamamoto K, Abedi K, Eslami P, Hernandez G, et al Dietary fish oil prevents vascular dysfunction and oxidative stress in hyperinsulinemic rats. Am J Hypertens. 2005;18(2 Pt 1):213–9. 10.1016/j.amjhyper.2004.08.030 15752949

[41] Tsuduki T, Honma T, Nakagawa K, Ikeda I, Miyazawa T. Long-term intake of fish oil increases oxidative stress and decreases lifespan in senescence-accelerated mice. Nutrition. 2011;27(3):334–7. 10.1016/j.nut.2010.05.017 20621447

[42] Roy S, Kale A, Dangat K, Sable P, Kulkarni A, Joshi S. Maternal micronutrients (folic acid and vitamin B(12)) and omega 3 fatty acids: implications for neurodevelopmental risk in the rat offspring. Brain Dev. 2012;34(1):64–71. 10.1016/j.braindev.2011.01.002 21300490

[43] Kasture V, Dalvi S, Swamy M, Kale A, Joshi S. Omega-3 fatty acids differentially influences embryotoxicity in subtypes of preeclampsia. Clin Exp Hypertens. 2020;42(3):205–12. 10.1080/10641963.2019.1601208 30964712

[44] Kemse NG, Kale AA, Joshi SR. A combined supplementation of omega-3 fatty acids and micronutrients (folic acid, vitamin B12) reduces oxidative stress markers in a rat model of pregnancy induced hypertension. PLoS One. 2014;9(11):e111902 10.1371/journal.pone.0111902 25405347 PMC4236044

[45] Barden AE, Mori TA, Dunstan JA, Taylor AL, Thornton CA, Croft KD, et al Fish oil supplementation in pregnancy lowers F2-isoprostanes in neonates at high risk of atopy. Free Radic Res. 2004;38(3):233–9. 10.1080/10715760310001656722 15129731

[46] Kajarabille N, Hurtado JA, Pena-Quintana L, Pena M, Ruiz J, Diaz-Castro J, et al Omega-3 LCPUFA supplement: a nutritional strategy to prevent maternal and neonatal oxidative stress. Matern Child Nutr. 2017;13(2). 10.1111/mcn.12300 PMC6865966 27072591

[47] See VH, Mas E, Burrows S, O’Callaghan NJ, Fenech M, Prescott SL, et al Prenatal omega-3 fatty acid supplementation does not affect offspring telomere length and F2-isoprostanes at 12 years: A double blind, randomized controlled trial. Prostaglandins Leukot Essent Fatty Acids. 2016;112:50–5. 10.1016/j.plefa.2016.08.006 27637341

[48] Bruschi M, Santucci L, Petretto A, Bartolocci M, Marchisio M, Ghiggeri GM, et al Association between maternal omega-3 polyunsaturated fatty acids supplementation and preterm delivery: A proteomic study. FASEB J. 2020;34(5):6322–34. 10.1096/fj.201900738RR 32162735

[49] Jones ML, Mark PJ, Mori TA, Keelan JA, Waddell BJ. Maternal dietary omega-3 fatty acid supplementation reduces placental oxidative stress and increases fetal and placental growth in the rat. Biol Reprod. 2013;88(2):37 10.1095/biolreprod.112.103754 23269667

[50] Jones ML, Mark PJ, Waddell BJ. Maternal omega-3 fatty acid intake increases placental labyrinthine antioxidant capacity but does not protect against fetal growth restriction induced by placental ischaemia-reperfusion injury. Reproduction. 2013;146(6):539–47. 10.1530/REP-13-0282 24023246

[51] Meher AP, Joshi AA, Joshi SR. Maternal micronutrients, omega-3 fatty acids, and placental PPARgamma expression. Applied physiology, nutrition, and metabolism. 2014;39(7):793–800. 10.1139/apnm-2013-0518 24749811

[52] Patten AR, Brocardo PS, Christie BR. Omega-3 supplementation can restore glutathione levels and prevent oxidative damage caused by prenatal ethanol exposure. J Nutr Biochem. 2013;24(5):760–9. 10.1016/j.jnutbio.2012.04.003 22841392

[53] Ramaiyan B, Bettadahalli S, Talahalli RR. Dietary omega-3 but not omega-6 fatty acids down-regulate maternal dyslipidemia induced oxidative stress: A three generation study in rats. Biochem Biophys Res Commun. 2016;477(4):887–94. 10.1016/j.bbrc.2016.06.153 27373826

[54] Roy S, Sable P, Khaire A, Randhir K, Kale A, Joshi S. Effect of maternal micronutrients (folic acid and vitamin B12) and omega 3 fatty acids on indices of brain oxidative stress in the offspring. Brain Dev. 2014;36(3):219–27. 10.1016/j.braindev.2013.03.004 23622878

[55] Garcia-Rodriguez CE, Helmersson-Karlqvist J, Mesa MD, Miles EA, Noakes PS, Vlachava M, et al Does increased intake of salmon increase markers of oxidative stress in pregnant women? The salmon in pregnancy study. Antioxid Redox Signal. 2011;15(11):2819–23. 10.1089/ars.2011.4108 21689025

[56] Olsen SF, Sørensen JD, Secher NJ, Hedegaard M, Henriksen TB, Hansen HS, et al Randomised controlled trial of effect of fish-oil supplementation on pregnancy duration. Lancet. 1992;339(8800). 10.1016/0140-6736(92)90533-9 1349049

[57] Olsen SF, Secher NJ, Tabor A, Weber T, Walker JJ, Gluud C. Randomised clinical trials of fish oil supplementation in high risk pregnancies. Fish Oil Trials In Pregnancy (FOTIP) Team. BJOG. 2000;107(3):382–95. 10.1111/j.1471-0528.2000.tb13235.x 10740336

[58] Szajewska H, Horvath A, Koletzko B. Effect of n-3 long-chain polyunsaturated fatty acid supplementation of women with low-risk pregnancies on pregnancy outcomes and growth measures at birth: a meta-analysis of randomized controlled trials. Am J Clin Nutr. 2006;83(6):1337–44. 10.1093/ajcn/83.6.1337 16762945

[59] Salvig JD, Olsen SF, Secher NJ. Effects of fish oil supplementation in late pregnancy on blood pressure: a randomised controlled trial. Br J Obstet Gynaecol. 1996;103(6):529–33. 10.1111/j.1471-0528.1996.tb09801.x 8645644

[60] Smuts CM, Huang M, Mundy D, Plasse T, Major S, Carlson SE. A randomized trial of docosahexaenoic acid supplementation during the third trimester of pregnancy. Obstet Gynecol. 2003;101(3):469–79. 10.1016/s0029-7844(02)02585-1 12636950

[61] Zhou SJ, Yelland L, McPhee AJ, Quinlivan J, Gibson RA, Makrides M. Fish-oil supplementation in pregnancy does not reduce the risk of gestational diabetes or preeclampsia. Am J Clin Nutr. 2012;95(6):1378–84. 10.3945/ajcn.111.033217 22552037

[62] da Silva Lopes K, Ota E, Shakya P, Dagvadorj A, Balogun OO, Pena-Rosas JP, et al Effects of nutrition interventions during pregnancy on low birth weight: an overview of systematic reviews. BMJ Glob Health. 2017;2(3):e000389 10.1136/bmjgh-2017-000389 29018583 PMC5623264

[63] Kar S, Wong M, Rogozinska E, Thangaratinam S. Effects of omega-3 fatty acids in prevention of early preterm delivery: a systematic review and meta-analysis of randomized studies. Eur J Obstet Gynecol Reprod Biol. 2016;198:40–6. 10.1016/j.ejogrb.2015.11.033 26773247

[64] Raatz SK, Redmon JB, Wimmergren N, Donadio JV, Bibus DM. Enhanced absorption of n-3 fatty acids from emulsified compared with encapsulated fish oil. J Am Diet Assoc. 2009;109(6):1076–81. 10.1016/j.jada.2009.03.006 19465191 PMC2701654

[65] Wilson NA, Mantzioris E, Middleton PT, Muhlhausler BS. Gestational age and maternal status of DHA and other polyunsaturated fatty acids in pregnancy: A systematic review. Prostaglandins Leukot Essent Fatty Acids. 2019;144:16–31. 10.1016/j.plefa.2019.04.006 31088623

[66] Brantsaeter AL, Haugen M, Hagve TA, Aksnes L, Rasmussen SE, Julshamn K, et al Self-reported dietary supplement use is confirmed by biological markers in the Norwegian Mother and Child Cohort Study (MoBa). Ann Nutr Metab. 2007;51(2):146–54. 10.1159/000103275 17536192

[67] Skarke C, Alamuddin N, Lawson JA, Li X, Ferguson JF, Reilly MP, et al Bioactive products formed in humans from fish oils. Journal of Lipid Research. 2015;56(9):1808–20. 10.1194/jlr.M060392 26180051 PMC4548785

[68] Oken E. Fish consumption and marine omega-3 fatty acid supplementation in pregnancy. In: Post TW, Barss V., editor. UpToDate: UpToDate; 2020.

[69] Zhang Z, Fulgoni VL, Kris-Etherton PM, Mitmesser SH. Dietary Intakes of EPA and DHA Omega-3 Fatty Acids among US Childbearing-Age and Pregnant Women: An Analysis of NHANES 2001–2014. Nutrients. 2018;10(4). 10.3390/nu10040416 PMC5946201 29597261

