# Supplementary material for: Omega-3 fatty acid supplement use and oxidative stress levels in pregnancy
Source: PLoS One. 2020 Oct 23;15(10):e0240244. doi: 10.1371/journal.pone.0240244 (PMC7584173; doi:10.1371/journal.pone.0240244)
Supplement: S1 Table — a. Model includes gestational age at sample collection, specific gravity, maternal age, race, education, and study center. (DOCX) [file pone.0240244.s001.docx]

**S1 Table.** Adjusted percent change (95% confidence intervals) in urinary oxidative stress levels in association with omega-3 fatty acid supplement use in the 3^rd^ trimester of pregnancy stratified by education level

|  | Finalized Model^a^ (n=693) | No College Degree  (n=176) | College Degree  (n=517) | p for interaction |
| --- | --- | --- | --- | --- |
| Measured |  |  |  |  |
| 8-iso-prostaglandin F_2α_ | -10.2 (-19.6, 0.25) | -23.1 (-55.0, 31.1) | -7.93 (-17.2, 2.39) | 0.51 |
| 8-iso-prostaglandin F_2α_ metabolite | -10.3 (-17.1, -2.91) | -29.8 (-52.2, 3.15) | -8.38 (-15.0, 1.19) | 0.09 |
| Prostaglandin F_2α_ | 1.91 (-11.2, 17.0) | -11.4 (-50.3, 58.0) | 3.07 (-10.7, 19.0) | 0.62 |
| Derived |  |  |  |  |
| 8-iso-prostaglandin F_2α_, enzymatic | 16.7 (-17.4, 64.8) | 22.4 (-80.7, 676.4) | 16.9 (-15.6, 62.0) | 0.93 |
| 8-iso-prostaglandin F_2α_, chemical | -18.7 (-30.1, -5.32) | -31.6 (-64.9, 33.5) | -15.6 (-27.5, 1.74) | 0.58 |

a. Model includes gestational age at sample collection, specific gravity, maternal age, race, education, and study center.
