# Supplementary material for: Omega-3 fatty acid supplement use and oxidative stress levels in pregnancy
Source: PLoS One. 2020 Oct 23;15(10):e0240244. doi: 10.1371/journal.pone.0240244 (PMC7584173; doi:10.1371/journal.pone.0240244)
Supplement: S2 Table — a. Model includes gestational age at sample collection, specific gravity, maternal age, race, education, and study center. (DOCX) [file pone.0240244.s002.docx]

**S2 Table.** Adjusted^a^ percent change (95% confidence intervals) in urinary oxidative stress levels in association with omega-3 fatty acid supplement use in the 3^rd^ trimester, restricted to women who used prenatal vitamins in 3^rd^ trimester

|  | All women (n=693) | 3^rd^ trimester prenatal vitamin users  (n=622) |
| --- | --- | --- |
| Measured |  |  |
| 8-iso-prostaglandin F_2α_ | -10.2 (-19.6, 0.25) | -8.33 (-46.3, 56.5) |
| 8-iso-prostaglandin F_2α_ metabolite | -10.3 (-17.1, -2.91) | -10.3 (-17.2, -2.84) |
| Prostaglandin F_2α_ | 1.91 (-11.2, 17.0) | -0.15 (-13.4, 15.1) |
| Derived |  |  |
| 8-iso-prostaglandin F_2α_, enzymatic | 16.7 (-17.4, 64.8) | 7.92 (-23.3, 51.9) |
| 8-iso-prostaglandin F_2α_, chemical | -18.7 (-30.1, -5.32) | -14.6 (-26.8, -0.33) |

a. Model includes gestational age at sample collection, specific gravity, maternal age, race, education, and study center
